# Receptor-Based Pharmacophore Modeling in the Search for Natural Products for COVID-19 M^pro^

**DOI:** 10.3390/molecules26061549

**Published:** 2021-03-11

**Authors:** Mohd Saeed, Amir Saeed, Md Jahoor Alam, Mousa Alreshidi

**Affiliations:** 1 Department of Biology, College of Sciences, University of Ha’il, Hail 2440, Saudi Arabia; j.alam@uoh.edu.sa (M.J.A.); mo.alreshidi@uoh.edu.sa (M.A.); 2 Department of Clinical Laboratory Sciences, College of Applied Medical Sciences, University of Ha’il, Hail 2440, Saudi Arabia; am.saeed@uoh.edu.sa

**Keywords:** COVID-19, main protease, receptor-based pharmacophore, molecular docking, natural compounds

## Abstract

Considering the urgency of the COVID-19 pandemic, we developed a receptor-based pharmacophore model for identifying FDA-approved drugs and hits from natural products. The COVID-19 main protease (M^pro^) was selected for the development of the pharmacophore model. The model consisted of a hydrogen bond acceptor, donor, and hydrophobic features. These features demonstrated good corroboration with a previously reported model that was used to validate the present model, showing an RMSD value of 0.32. The virtual screening was carried out using the ZINC database. A set of 208,000 hits was extracted and filtered using the ligand pharmacophore mapping, applying the lead-like properties. Lipinski’s filter and the fit value filter were used to minimize hits to the top 2000. Simultaneous docking was carried out for 200 hits for natural drugs belonging to the FDA-approved drug database. The top 28 hits from these experiments, with promising predicted pharmacodynamic and pharmacokinetic properties, are reported here. To optimize these hits as M^pro^ inhibitors and potential treatment options for COVID-19, bench work investigations are needed.

## 1. Introduction

There is currently no available medication for the treatment of COVID-19. A number of vaccines and drug molecules are in clinical trials, but none of the drug molecules are available at the time of writing [1]. National regulatory agencies are evaluating certain COVID-19 vaccinations, and one has been approved in some countries. Wide trials of multiple vaccine candidates have shown promising preliminary findings, and more candidates will likely be sent to the regulatory authorities for approval. There are several possible candidates for the COVID-19 vaccine currently in production. The WHO works with international allies to help coordinate crucial measures in this system, and to promote equal access to secure and reliable COVID-19 vaccines for the millions of people who need it. As there is still no proven viable treatment for the disease, with daily and linear changes, the incidence of new cases tends to increase. The pandemic has caused more than 83 million infections and more than 1.8 million deaths as of the last week of December 2020. With the onset of the pandemic, the need to pursue an efficient treatment is now more urgent than at any other moment. Scientists from various fields of expertise around the world are seeking to identify the most effective treatments, whether they are already proven to cure other diseases or are novel compounds (synthetic/natural), that can address the SARS-CoV2 mechanism by which the virus attacks and replicates in human cells [2].

Structural proteins (e.g., spike protein, membrane protein, angiotensin-converting enzyme 2 (ACE2), etc.) and non-structural proteins (of which there are 16 in total; e.g., main protease (M^pro^), papain-like protease, helicase, etc.) constitute several known drug targets [3,4]. From the perspective of drug design, the main structural and non-structural proteins could play important roles [5]. ACE2 is a key SARS-CoV-2 receptor target that plays a critical role in disease pathogenesis since it makes viral entry into the target cells [6].

It is well documented that the M^pro^ of SARS-CoV-2 is one of the most tempting drug targets, as viral maturation eventually depends mainly on the function of M^pro^ [7,8,9]. The inhibition of M^pro^ has been shown to obstruct viral replication in certain studies. In several early and recent studies targeting M^pro^, numerous novel inhibitors have been produced against this enzyme—either novel compounds or existing drugs [9]. Blocking M^pro^’s activity would therefore prevent viral replication and transcription. Moreover, it is recognized that no proteases with a similar cleavage specificity are present in humans, so inhibitors are more likely to be non-toxic [10]. Targeted screening of natural compounds could therefore be an alternative for the discovery of a possible M^pro^ inhibitor for SARS-CoV-2.

For this purpose, computational methods such as pharmacophore-based virtual screening and molecular docking simulation can be utilized efficiently [11]. The pharmacophore model describes the spatial arrangement of groups with respect to the chemical features of the active site [12]. These features can be assembled to select features for obtaining a structure-based pharmacophore (SBP). SBP modeling can be macromolecule–ligand complex-based or macromolecule-based (without a ligand) [13]. The ligand bind within a receptor can be used to develop a meaningful pharmacophore that can be used as a query for virtual screening.

In this study, the pharmacophore model was followed for a receptor-based ligand by interaction generation using LUDI. A component of the Discovery Studio suite, LUDI is a computer program that places small molecules at the active protein site so that the enzyme can form hydrogen bonds, and hydrophobic pockets are filled with hydrophobic groups. When the 3D structure of the protein-inhibitor complex is known, the LUDI protocol is often used to suggest new substituents for an already known inhibitor. LUDI can fit fragments into interaction sites and link them to an existing ligand at the same time [14,15,16]. LUDI generates the features that may be tedious to detect in virtual screening and other experiments, such as in ligand-based pharmacophore modeling [15,17]. The next step involved the transformation of interactions using the pharmacophoric features available in the Catalyst program, i.e., using various features such as the H-bond acceptor, H-bond donor, and a hydrophobe. The resulting features from the SBP were correlated to ligand features for the development of a model of features based on proteins critical for binding [17]. Using these computational approaches, a range of potential natural compounds were identified in this work that could prove to be possible drug compounds after the wet lab and clinical studies.

## 2. Results

### 2.1. LUDI Receptor-Based Pharmacophore Generation

The LUDI-based pharmacophore was developed using state-of-the-art techniques in pharmacophore modeling. The pharmacophore consisted of a hydrogen bond acceptor, a hydrogen bond donor, and hydrophobic features. The model is valid and corroborates our previously reported model for M^pro^. This protocol validates our previous study on implementation in pharmacophore-based virtual screening experiments (Supplementary Figure S1) [9].

**Figure 1:**
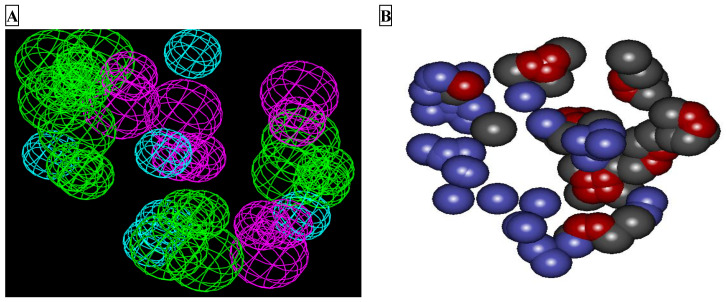
Feature mapping of the binding site of M^pro^ (PDB ID: 6LU7). (**A**) LUDI-based pharmacophore model developed in Discovery Studio showing hydrophobic features (sky blue), hydrogen bond acceptor (green), and hydrogen bond donor (violet). (**B**) Molegro-based features developed for the validation of pharmacophores developed by LUDI: stearic (blue), hydrophobic (carbon color), and electronic (red) features.

The protocol for the development of the receptor-based pharmacophore model has been reported in the literature. The model developed through LUDI was revalidated here using Molegro Virtual Docker (MVD) 6.0 [18]. The developed template was then used as a query for validation of the developed features of the LUDI-based model. The LUDI-based model for the pharmacophore is presented in Figure 1A, while the model developed through MVD 6.0 is presented in Figure 1B.

### 2.2. Pharmacophore Validation

**Figure 2:**
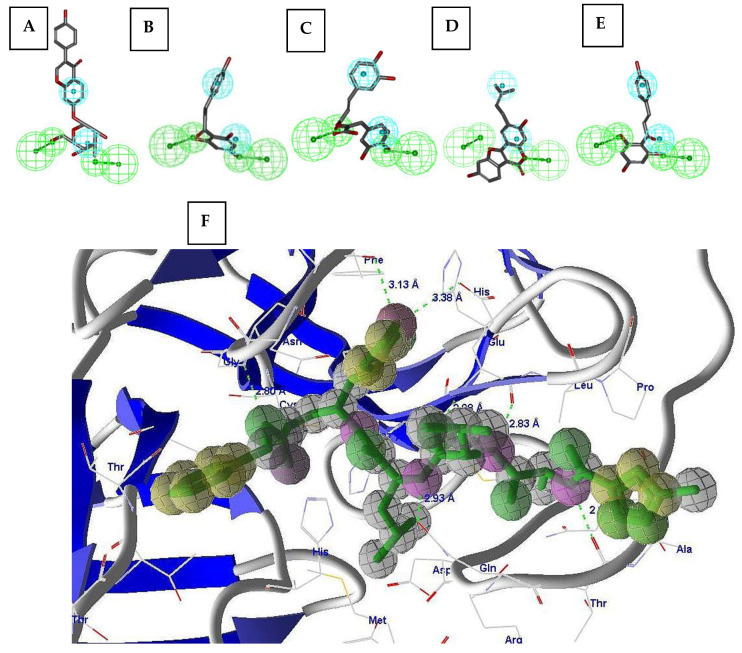
(**A**–**E**) The ligand pharmacophore mapping of (**A**) daidzin, (**B**) phloretin, (**C**) rosmarinic acid, (**D**) psoralidin, and (**E**) naringenin chalcone. (**F**) The representative figures for the simplified pharmacophore model developed in LUDI for PDB 6LU7. The features extracted by MVD 6.0, the reported pharmacophore model, and the LUDI are the same. The RMSD of 0.032882 Å between the two models also justified this finding. Red, donor; green, acceptor; yellow, hydrophobic features.

The model developed in LUDI was validated using the reported pharmacophore model. MVD 6.0 was used for developing the template and identifying the features of the co-crystal ligand from the 6LU7 in order to compare them with those from LUDI. The MVD model developed well-validated features of the LUDI model as hydrophobic (yellow), stearic (green), and electronic (red) features, respectively. The mappings in Figure 2A–E explored the potential of the drugs identified through LUDI in the reported model of the pharmacophore. They clearly show that the identified drugs mapped well on the reported pharmacophore model, thus cross-verifying the model developed in LUDI and demonstrating its potential for virtual screening. Figure 2F presents the features developed in 6LU7 using MVD 6.0. This model was also authenticated using the previously reported model for M^pro^ with well-defined training and a test set of compounds [9]. The RMSD value of 0.032882 Å indicated the robustness of the model for virtual screening experiments.

### 2.3. Virtual Screening

The pharmacophore developed through LUDI and MVD 6.0 was used for the virtual screening of the database of natural resources. The ZINC database was screened for the FDA-approved drugs and leads belonging to the natural products class. A total of 208,000 compounds were prepared from the database and used for the virtual screening experiments. The top 20,000 compounds were prioritized from these. Out of these, 2000 compounds were selected for further validation using the LUDI-based model, and out of these, the top 200 were used for further docking experiments.

The identified leads were mapped on the previously reported model for M^pro^ [9]. These FDA-approved drugs and leads from the database were prioritized based on the fit value filter for both of the models presented in Table 1. Lipinski’s rule of five and other rules were applied for the identified ligands in the database preparation so that there would be a lesser chance of failure of the drug.

### 2.4. Molecular Docking Experiments

The molecular docking studies were carried out using MVD 6.0. The previously reported protocol was used for the docking runs. The model was validated using the redocking of the co-crystal ligand in the binding site of the protein. These scores were taken as reference scores for the identified leads through virtual screening. The binding site was assigned against the active site residues, namely Phe140, Asn142, Gly143, Cys145, His163, His164, Met165, Glu166, Gln189, and Thr190 for PDB ID 6LU7. The binding affinity was also considered for the interaction analysis to determine the probable effect on M^pro^ or 3CLpro for the 6LU7.

The three parameters considered for the results were MolDock, rerank, and docking, and similarity scores for the compounds identified via virtual screening are shown in Table 1. The most promising natural products from this screening were identified and are reported in the table. Previous studies and internal standard molecules tested with in vitro experiments for the targeted activity were also taken into consideration for the prioritization of compounds from the database screening. The top active molecule from the dataset was analyzed for its interactions, through hydrogen bond interactions, with conserved residues in the binding site of M^pro^ and for hydrogen bond interactions (HBIs) with the amino acids Ser143, Gly144, Leu141, His164, His165, and Cys145. Similarly, the subsequent ligands interacted with His164 in addition to these interactions. The hydrophobic interactions of the identified leads were observed with the amino acids His163, His164, Met165, Glu166, Gln189, and Thr190.

The possible reasons for the higher binding scores of these compounds are the interactions with important amino acids and the hydrophobic interactions of these molecules with amino acids. This was taken into consideration for the prioritization of leads. The identified natural products were further analyzed by the pharmacokinetic parameters in order to provide potential treatment prospects for the pandemic.

### 2.5. Molecular Docking of Prioritized Drugs

**Figure 3:**
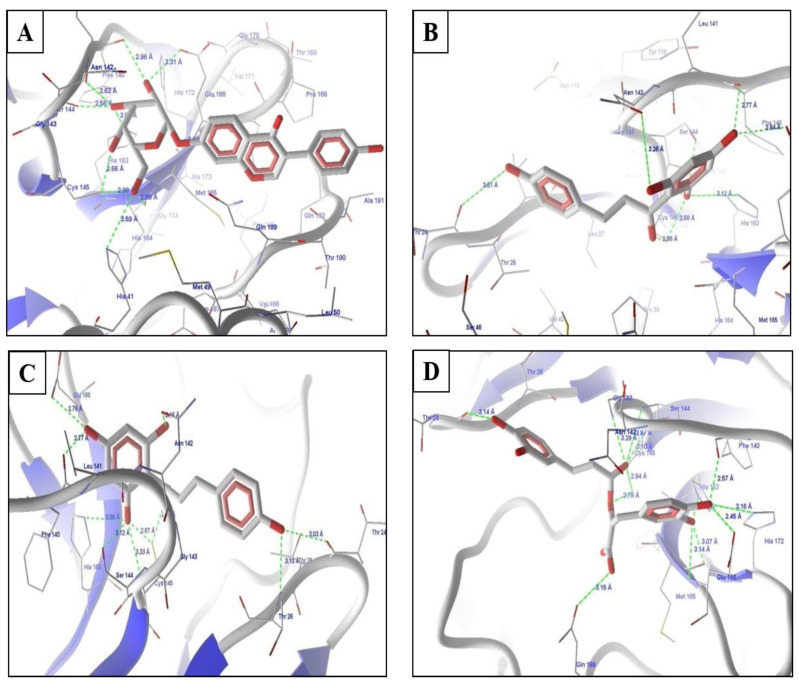

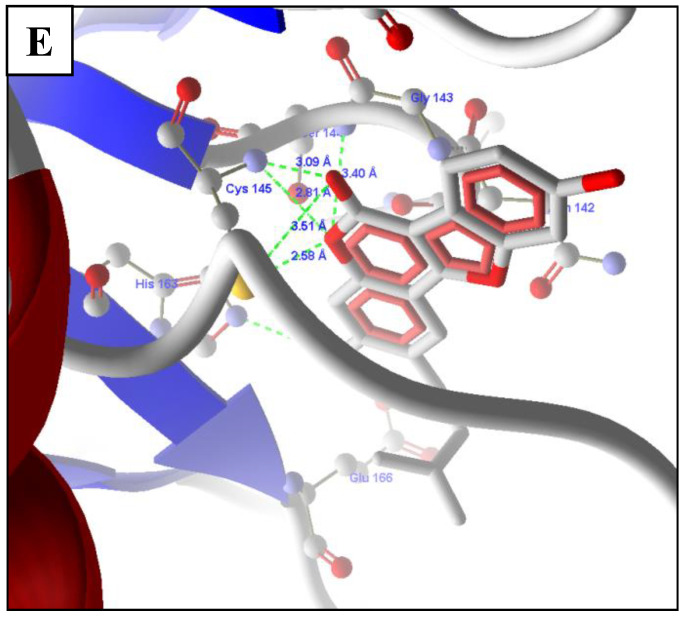
Molecular interactions with the top hits from the virtual screenings of (**A**) daidzin, (**B**) naringerin, (**C**) phloretin, (**D**) rosmeric acid, and (**E**) psoralidin.

The top predicted compounds from the pharmacophore-based virtual screening (PBVS) were docked in the protein M^pro^ using the default parameters and the binding site, as defined in the docking of all compounds used in the development of the pharmacophore model. The scores from the docking experiments of the natural products list are shown in Table 1. The reported pharmacophore model was used as a query for the virtual screening and the flexible fit was implemented for the same. The top-scoring drugs of this screening were daidzin, phloretin, and rosmarinic acid (Figure 3), which showed MolDock scores of 115.112, −113.173, and −130.853 and rerank scores of −111.544, −90.7301, and −103.821, respectively. Psoralidin was also identified as a potential lead. Psoralidin, the use of which has been previously reported in relation to leprosy, also showed good interactions, with a viral M^pro^ MolDock score of −115.765 and rerank score of −92.1257 (Figure 3).

### 2.6. Revalidation of Docking Using Autodock

The top-five screened compounds, rosmarinic acid, higenamine hydrochloride, phloretin, daidzin, and naringenin chalcone, were further docked against the active site residues of 6LU7 using Autodock. It was found that the binding affinities of these compounds were highly similar to those found with MolDock. The top-scoring compound from MolDock, rosmarinic acid (MolDock score = −130.853), had the highest binding energy in Autodock (−7.6 Kcal/mol), and other compounds showed similar trends: higenamine hydrochloride, phloretin, daidzin, and naringenin chalcone resulted in −7, −6.7, −6, and −5.9 Kcal/mol, respectively. The binding energy of psoralidin showed a good corroboration with −115.765 anda rerank score of −92.1257.

### 2.7. Physicochemical Parameters for the Selected Ligands

The physicochemical parameters for the identified ligands were identified using an online tool, Swiss ADME [19]. The leads identified through this study were therefore found suitable for further study, as they qualified the pharmacokinetic and absorption, distribution, metabolism, and toxicity parameters, as demonstrated by the data shown in Table 2.

## 3. Discussion

The pharmacophore features were compared with the reported model of the M^pro^ and the comparison explored the validity and the potential of the model in the virtual screening experiments. The pharmacophore mimicked the hypothetical receptor site in 3D space and LUDI showed the same in the receptor. Figure 2A–E showed the mapping of the molecules in the pharmacophore along with the LUDI- and MVD-based modeling of the features in the receptor. Active site residues, such asPhe140, Asn142, Gly143, Cys145, His163, His164, Met165, Glu166, Gln189, and Thr190, for PDB ID 6LU7 were considered for the development of the model using LUDI. The developed model was well-validated by the reported model. The model was iterated for virtual screening experiments using state-of-the-art techniques reported in the literature [9,11].

Daidzin (Figure 3A) showed hydrogen bond interactions with amino acids in the binding site, namely Ser144, His163, Glu166, and Cys145. The molecule also showed hydrophobic interactions with His163, His164, Met165, Glu166, Gln189, Thr190, and Glu192. The side chains showed hydrophobic interactions with these amino acids, while the parent daidzin nucleus with –NH functionality showed hydrogen bond interactions and additional hydrophobic interactions with amino acids such asPhe140. The higher binding scores of this drug were due to higher hydrogen bond interactions and hydrophobic interactions in the binding sites of the target proteins. Daidzin as isoflavone is an active and selective inhibitor of human mitochondrial aldehyde dehydrogenase and has also been identified as an antiviral agent in recent studies [20,21,22].

The next drug from the database approved as a natural product was phloretin (Figure 3B), which binds with the target protein. Phloretin has been reported as an effective antiviral against the Zika virus [23] and has been identified as showing antioxidant, anti-inflammatory, and anticarcinogenic activities, among others [24]. Phloretin showed a cascade of hydrogen bond interactions with Leu141, Ser144, Cys145, His163, and Glu166. The molecule formed good hydrophobic contacts with the amino acids Thr26, Phe140, Pro168, Met165, Thr190, Gln189, and Asn142. The aromatic rings of phloretin form a butterfly-like structure in the binding site and thus have hydrophobic interactions with amino acids. The methyl piperazine ring also showed additional hydrophobic interactions.

The next identified drug from screening was rosmarinic acid (Figure 3C), which showed HBIs with Leu141, Gly143, Ser144, Cys145, and His163. Rosmarinic acid has a variety of known properties, including anti-inflammatory, anticyclooxygenase, anti-oxidant properties, and has shown effective use as a hepatitis B virus inhibitor [25]. It showed hydrophobic interactions with Thr26, Thr24, Thr25, Met165, and Phe140. The higher scores are representative of the good binding affinity of this drug for M^pro^. The representative scores of the identified hits from the natural product library are shown in Table 1.

The last, important compound for the target was psoralidin, which showed HBIs with Leu141, Gly143, Ser144, Cys145, and His163. It also showed similar hydrophobic and electrostatic interactions with amino acids in the binding site. The higher scores were due to their interactions with important amino acids in the binding site with reference to standard compounds. Psoralidin has anticancer, antiosteoporotic, anti-inflammatory, antivitiligo, antibacterial, antiviral, and antidepressant-like properties, as per growing preclinical proof [26].

## 4. Material and Methods

### 4.1. LUDI-Based Pharmacophore Model

The pharmacophore model based on the binding site of the target proteins was developed using state-of-the-art pharmacophore modeling techniques, as stated earlier [9,27,28]. In the present scenario, the co-complexed structures were used to develop the pharmacophore model using the reported crystal structure 6LU7 [29]. The novel model was based on a receptor-based pharmacophore model using LUDI [30].

### 4.2. Common Feature Pharmacophore Generation

An earlier reported pharmacophore model was used to compare the developed model through LUDI. The pharmacophore generation protocol was performed using the HipHop algorithm of Catalyst, employed in Discovery Studio 2020 (DS 2020) [9]. The co-complexed ligand with protein in its original conformation was extracted to develop a pharmacophore model using DS 2020 Windows. The remaining protocol for the development and validation of the model has been previously reported [31]. The conformations of these compounds were generated using the ”diverse conformation generation” protocol of DS 2020, using default parameters (principal value = 2; maximum omit feature = 0; interfeature distance = 2Å).

### 4.3. Pharmacophore-Based Virtual Screening

The compounds available in a selected database (the ZINC database of natural products listed as FDA drugs) were imported in df format into Discovery Studio 2020 and processed using the tool “Prepare Ligands” [32,33]. The pharmacophore model developed with the co-complexed ligand with protein in its bioactive conformation was used as a query to identify the potential leads from this database of natural compounds, using the “Best Flexible Search” option in DS 2020. The resulting hits were screened based on fit values < 2.5, followed by additional screening using physiochemical properties. A set of 208,000 hits was produced and then filtered according to their lead-like properties. Lipinski’s filter was used to reduce hits to the top 20,000, and these were further reduced to 2000 with fit values based on the model. Out of these, the top 200 hits were selected for molecular docking. The PBVS approach was used to identify potential hits for COVID-19. Twenty-eight of these top hits were selected, based on the MolDock score and re-rank scores, for further study.

### 4.4. Molecular Docking Studies

The molecular docking studies were performed using the MVD software [18]. The respective MolDock and rerank scores were iterated using piecewise linear potentials (PLPs) [34]. PDB ID 6LU7 [35] was used and prepared according to the reported protocol. A population size of 50 and a maximum number of iterations of 1500 were set as parameters.

## 5. Conclusions

This study resulted in the identification of natural products available for virtual screening from a database. The molecules identified from the screening showed a good binding affinity towards active sites of the M^pro^. The binding site also showed good scores as compared to the standard ligands and our previously reported leads from a study in this area. Daidzin, phloretin, rosmarinic acid, higenamine hydrochloride, and naringenin chalcone were anticipated to be effective natural inhibitors of M^pro^. The top-scoring compound from MolDock, rosmarinic acid (MolDock score = −130.853), showed the highest binding energy in Autodock (−7.6 Kcal/mol), and other compounds showed similar trends: higenamine hydrochloride, phloretin, daidzin, and naringenin chalcone resulted in −7, −6.7, −6, and −5.9 Kcal/mol, respectively. Although it is too early to draw conclusions based on our study alone, it is hoped that the identified natural products might prove to have therapeutic uses against the deadly pandemic.

## Figures and Tables

**Table 1:** Scores of the MolDock run.

Name of the Natural Product	MolDock Score	Rerank Score	Docking Score	Similarity Score	Fit Value ^a^
Daidzin	−115.112	−111.544	−362.165	−248.271	2.94536
Phloretin	−113.173	−90.7301	−286.075	−170.686	3.1189
Rosmarinic acid	−130.853	−103.821	−361.153	−225.21	3.08619
Higenamine hydrochloride	−115.15	−98.3765	−257.79	−142.498	3.16578
Psoralidin	−115.765	−92.1257	−316.84	−196.709	3.122
Naringenin chalcone	−118.833	−95.914	−270.535	−137.061	2.96379
Pinoresinol dimethyl ether	−118.818	−85.9625	−312.815	−195.726	3.16578
AMPHICOL (chloramphenicol)	−77.3877	−62.1465	−218.843	−141.977	2.90662
Aloe-emodin	−97.7624	−87.7886	−234.279	−135.969	3.2435
Caffeic acid phenethyl ester (CAPE)	−101.871	−85.2197	−312.47	−211.714	2.95378
Dopamine hydrochloride	−69.3166	−58.7612	−176.013	−107.296	3.16578
Epinephrine bitartrate	−77.6416	−64.9263	−205.116	−126.866	2.96379
Genipin	−102.454	−82.0033	−221.112	−114.184	3.26523
Morin	−105.509	−92.3222	−278.217	−165.081	3.00264
Noradrenaline bitartrate	−74.4106	−61.1462	−192.769	−119.856	3.45066
Scopoletin	−88.1501	−74.2017	−195.512	−102.584	2.95378
N-Sulfo-glucosamine	−92.1255	−68.4776	−214.491	−97.6027	2.94536
Nordihydroguaiaretic acid	−100.616	−71.7295	−297.163	−196.406	2.96379
DL-Panthenol	−79.5991	−68.3015	−158.414	−72.983	3.26523
Oxyresveratrol	−94.9673	−79.3005	−259.463	−161.834	3.00264
Danshensu	−70.6003	−62.9828	−199.499	−130.037	3.45066
3-Indolepropionic acid	−91.4718	−71.9077	−215.996	−123.141	3.1189
Pyridoxal 5-phosphate	−78.5887	−65.6728	−219.155	−129.277	3.08619
2′-deoxyuridine	−88.4843	−75.9807	−225.239	−131.446	2.90662
D-Glucose 6-phosphate	−76.5959	−58.835	−191.605	−110.676	3.2435
Cadaverine	−55.1465	−45.497	−95.4426	−40.6983	2.95378
Ethyl caffeate	−95.3421	−80.9775	−209.814	−115.739	2.94536
Sesamin	−102.973	−72.0989	−337.484	−235.388	3.26523
XYLO-PFAN (xylose)	−70.0215	−55.1009	−138.678	−55.264	3.00264

^a^ The fit values from the reported pharmacophore model [9] based on the ligand pharmacophore mapping protocol using the flexible fit method.

**Table 2:** Physicochemical properties of selected compounds.

Molecule		Daidzin	Phloretin	Rosmarinic Acid	Higenamine Hydrochloride	Naringenin Chalcone
Physicochemical Properties	Formula	C21H20O9	C15H14O5	C18H16O8	C16H18ClNO3	C15H12O5
	MW	416.38	274.27	360.31	307.77	272.25
	Heavy atoms	30	20	26	21	20
	Aromatic heavy atoms	16	12	12	12	12
	Fraction Csp3	0.29	0.13	0.11	0.25	0
	Rotatable bonds	4	4	7	2	3
	H-bond acceptors	9	5	8	4	5
	H-bond donors	5	4	5	4	4
	MR	104.09	74.02	91.4	88.11	74.34
	TPSA	149.82	97.99	144.52	72.72	97.99
Pharmacokinetics	GI absorption	Low	High	Low	High	High
	BBB permeant	No	No	No	Yes	No
	Pgp substrate	No	No	No	Yes	No
	CYP1A2 inhibitor	No	Yes	No	No	Yes
	CYP2C19 inhibitor	No	No	No	No	No
	CYP2C9 inhibitor	No	Yes	No	No	Yes
	CYP2D6 inhibitor	No	No	No	Yes	No
	CYP3A4 inhibitor	No	Yes	No	Yes	Yes
	log Kp (cm/s)	−8.36	−6.11	−6.82	−6.01	−5.96
Druglikeness	Lipinski violations	0	0	0	0	0
	Ghose violations	0	0	0	0	0
	Veber violations	1	0	1	0	0
	Egan violations	1	0	1	0	0
	Muegge violations	0	0	0	0	0
	Bioavailability score	0.55	0.55	0.56	0.55	0.55
Medicinal Chemistry	PAINS alerts	0	0	1	1	0
	Brenk alerts	0	0	2	1	1
	Leadlikeness violations	1	0	1	0	0
	Synthetic accessibility	5.01	1.88	3.38	2.7	2.56

## Data Availability

Not applicable.

## Supplementary Materials

The following are available online: Figure S1: Reported model for Mpro inhibitors through Pharmacophore modeling [9].
