# Supplementary material for: Receptor-Based Pharmacophore Modeling in the Search for Natural Products for COVID-19 Mpro
Source: Molecules. 2021 Mar 11;26(6):1549. doi: 10.3390/molecules26061549 (PMC8000608; doi:10.3390/molecules26061549)

Supporting Information

Figure S1: Reported model for Mpro inhibitors through Pharmacophore modeling [9].

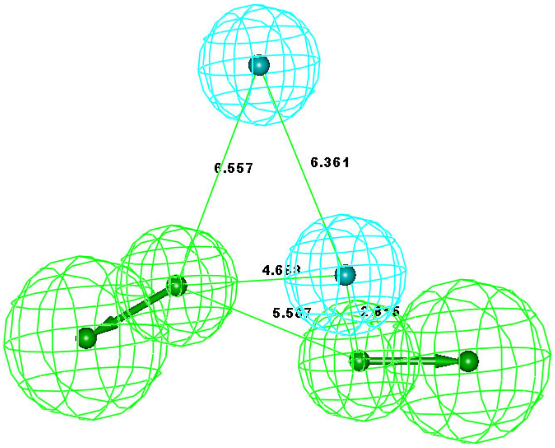

Supplement: Supplementary file 1 [file molecules-26-01549-s001.pdf]
